# The Diagnostic Sensitivity for Ulnar Neuropathy at the Elbow Is Not Increased by Addition of Needle EMG of ADM and FDI When Nerve Conduction Studies Are Normal

**DOI:** 10.3389/fneur.2019.00196

**Published:** 2019-03-11

**Authors:** Anne Kurver, Joost Smolders, Wim I. M. Verhagen, Jan Meulstee, Frouke A. P. Nijhuis

**Affiliations:** ^1^Department of Neurology, Canisius Wilhelmina Hospital, Nijmegen, Netherlands; ^2^Department of Clinical Neurophysiology, Canisius Wilhelmina Hospital, Nijmegen, Netherlands

**Keywords:** ulnar nerve entrapment, nerve conduction studies, diagnostic value, needle EMG, ulnaropathy

## Abstract

**Introduction:** The main objective of this study was to investigate whether electromyography (EMG) has additional value in the confirmation of the clinical diagnosis of ulnar nerve entrapment at the elbow (UNE) if nerve conduction studies (NCS) are normal.

**Methods:** A prospective cross-sectional cohort observational study was conducted among patients with the clinical suspicion of UNE. A total of 199 arms were included, who were examined according to a standard neurophysiological protocol, i.e., NCS and EMG relevant to the ulnar nerve.

**Results:** NCS were normal in 76 (38.2%) arms. No abnormal spontaneous muscle fiber activity was found with EMG in any of these cases. In 9 arms with normal NCS (11.8%), isolated abnormal MUAP configurations were found with EMG. Of these nine arms one UNE was diagnosed clinically, in which additional ultrasound and repeated NCS/EMG were negative. One had already been diagnosed with neuralgic amyotrophy and one with CTS. The other 6 arms had additional diagnostics which did not reveal an UNE.

**Conclusion:** EMG as part of the standard neurophysiological protocol exclusively in the confirmation of the clinical diagnosis of UNE has limited added value if NCS are normal in a high prior-odds setting. However, removing EMG may prevent detecting concomitant and/or additional differential diagnoses.

## Introduction

Ulnar nerve entrapment at the elbow (UNE) is the second most prevalent entrapment neuropathy, either at the cubital tunnel or at the epicondylar groove (1). An incidence of 18.9-25.2/100.000 person-years has been reported (2, 3). In medical history and neurological examination, numbness of the fifth digit and ulnar half of the fourth digit, weakness and atrophy of the hypothenar muscle group (e.g., abductor digiti minimi (ADM), the interosseous, and adductor pollicis muscles) may be found. The clinical diagnosis of UNE is usually not difficult, neurophysiological examination is often used for confirmation. Surgery for UNE can be highly effective; a previous study showed that 65–70% of patients who underwent UNE surgery, had a good to excellent recovery (4). In carpal tunnel syndrome (CTS) the majority of surgeons rarely operate without electrodiagnostic confirmation (5), which is probably also the case in UNE. In that case, it is important to have a good neurophysiological protocol for the confirmation of UNE.

The current standard neurophysiological protocol is well defined and effective in confirming the clinical diagnosis of UNE. For patients, however, the full protocol may be demanding, since electric stimulation and concentric needle-examination are often regarded as painful. Healthy volunteers scored the pain with a supramaximal-stimulated nerve conduction studies (NCS) of the median nerve on a numerical scale (0–10) with an average of 3.5 but a maximum of 7.0 (6). In children, EMG examination is often regarded as painful as venapuncture (7). Examiner experience, gender, reported pain tolerance, pain on NCS and earlier EMG studies did not predict the amount of pain experienced during the examination (8). Therefore, a good neurophysiological protocol should be complete, but also efficient.

Focal conduction slowing and focal partial conduction block may confirm the clinical diagnosis of UNE. In most laboratories, electromyography (EMG) of relevant muscles is performed as part of the standard protocol. However, in our experience, if carefully performed conduction studies show normal test results, EMG does not reveal relevant abnormalities. Therefore, we studied prospectively whether it is justified to refrain from EMG in these cases.

## Materials and methods

### Study Design and Setting

A prospective cross-sectional study was conducted among new patients (age > 18 years), who were referred to our department in 2016 for neurophysiological confirmation of the clinical suspicion of UNE. The criteria for UNE are sensory disturbance in the ulnar half of the fourth and fifth finger as well at the palmar or dorsal aspect of the hand and/or weakness of hand muscles innervated by the ulnar nerve (4). They were analyzed following standardized clinical procedures. When patients had bilateral complaints, both arms were included. In most patients, EMG was performed, except for a minority of cases in which patients did not give their consent. We aimed to investigate the neurophysiological protocol for confirmation of UNE, not to differentiate between diagnoses. After neurophysiological examination, all patients were referred back to their doctor to determine further examinations or the best therapy. The regional medical ethical commission (CMO Nijmegen-Arnhem, registration number: 2017–3129) and the hospital medical ethical commission (LTC) approved this research project. Since anonymized data were collected of patients receiving standard care, and no experimental procedures were applied, no informed consent was deemed necessary by these ethical committees.

### Neurophysiological Protocol

We performed a standard neurophysiological protocol, following the American Association of Electrodiagnostic Medicine (AAEM) criteria (9).

Motor and sensory NCS of the ulnar nerve were performed. The ulnar nerve was stimulated at the following sites: wrist, distal and proximal of the elbow and at the bicipital sulcus. Conduction distances were measured with a tape-measure with an accuracy of 5 mm. The only conduction distance with predetermined length was across the elbow (8 cm) (10). During all conduction studies, the elbow was kept flexed at 90 degrees. Stimulation was performed supramaximally. Compound muscle action potentials (CMAP) were recorded with surface electrodes. Sensory nerve action potentials (SNAP) were recorded antidromically using ring electrodes positioned around the fifth finger with an electrode distance of 4 cm, but less in smaller fingers (range 2–4 cm). CMAP and SNAP amplitudes were measured from negative to positive top in mV and μV, respectively. All latencies were measured from stimulus to onset deflection from baseline. Special care was taken to find the optimal positions of the “active” recording electrode above the hypothenar and first dorsal interosseal space (FDI) by shifting its position during stimulation, in order to achieve a maximal amplitude and an initial deflection that was as sharp as possible. During the whole procedure, CMAP configuration was observed in order to minimize or, if necessary, correct an altered position of the hand. The conduction velocity of all segments was computed. Prior to all tests, the arm was warmed by warming pads (11). Target temperature was 34°C, with a lower limit of 30°C. Skin temperature was monitored by an infrared thermometer device. Cooled regions were warmed again.

We used concentric needle electrodes (37 mm × 26G) to perform electromyography (EMG). FDI and ADM muscles were examined. More proximal muscles innervated by the ulnar nerve were not examined, since our protocol is only used for confirmation of UNE. At several sites within the muscle, the presence, and density of positive sharp waves and fibrillation potentials were determined carefully. The configuration of the motor unit action potentials (MUAPs), were examined visually. MUAPS were evaluated on amplitude and duration and whether they were polyphasic. Amplitude was considered high if majority of MUAPs was higher than 2 mV and amplitude was considered decreased when majority of MUAPS was below 200 mV. Normal range of duration was set at 5–15 ms, (screen set at 10 ms/div), increased duration was determined as more than 2 divisions. If the estimated percentage of polyphasic MUAPs was above 20%, this was classified as increased polyphasia.

For the interpretation of the electrodiagnostic findings, we used the AAEM criteria for UNE. A loss of sensory or motor conduction velocity of 10 m/s over the elbow, or a 20% loss of CMAP amplitude over the elbow, confirms UNE (9). The absence of the measured SNAP and CMAP responses is also an indication of UNE, however further examination is necessary to localize the definite site of the lesion.

### Statistics

Descriptive statistics are provided as mean number with corresponding standard deviation or number with corresponding percentages as appropriate. Missing data were noted. All analyses were conducted using SPSS (Statistical Package for Social Sciences, Chicago, IL, USA).

## Results

One hundred ninety-nine arms of 166 subjects [92 (46%) right and 107 (54%) left] were included between January 2016 and December 2016. The study population consisted of 74 men (45%). The mean age of patients was 54.5 ±14.5 years (range 19–91). Nine patients did not give their consent for EMG, and six patients did not give their consent for investigation of 2 muscles.

In 76 arms (38.2 %) NCS were normal. In these patients, EMG never showed abnormal spontaneous muscle fiber activity. In 48 of these 76 arms (63.2%) additional diagnostics were performed (Table 1). The following diagnoses were found in 74 patients (two missing diagnoses): EMG negative UNE (10.8%, confirmed with ultrasound), C7 radiculopathy (1.4%), C8 radiculopathy (4.1%), brachial plexus lesion (1.4%), myelopathy (1.4%), other neurological diagnosis (24.3%, quite often patients were diagnosed with CTS). In 56.8% of the patients with normal NCS/EMG, a neurological diagnosis was not found.

**Table 1:** Additional diagnostics (only when NCS was normal *n* = 76).

	**Included arms with normal NCS *n* (%)**	**Alternative diagnosis confirmed with additional diagnostics *n* (%)**	**UNE confirmed with ultrasound *n* (%)**
Total additional diagnostics	48/76 (63.2)	23/76 (30.3)	
Additional ultrasound for UNE	17/76 (22.4)		1/17 (5.9)
Additional MRI	15/76 (19.7)	10/15 (66.7)	
Additional NCS/EMG	28/76 (36.8)	11/28 (39.3)	

*NCS, nerve conduction studies; EMG, electromyography; UNE, ulnar nerve entrapment at the elbow*.

Of the 123 arms with abnormal results in NCS in which EMG was performed (*n* = 116), 27 arms (23.3%) showed abnormal spontaneous muscle fiber activity. Increased percentage of polyphasic MUAPs was found in 33 (28.4%) arms (Table 2). Clear differences between FDI and ADM were not found. Abnormalities were often found in only one of the two muscles. In 48.1% of the arms that showed abnormal spontaneous muscle fiber activity, and in 33.3% of the arms with an increased percentage of polyphasic MUAPs, it was found in both muscles.

**Table 2:** NCS results vs. EMG results included arms with available EMG data (*n* = 190).

	**Abnormal spontaneous muscle activity (FP±PSW) *n* (%)**	**Increased polyphasia *n* (%)**	**High Amplitude MUAPs *n* (%)**	**Long duration MUAPs *n* (%)**
Normal nerve conduction studies *N* = 74	0	4 (5.4)	3 (4.1)	3 (4.1)
Only abnormal motor conduction studies *N* = 48	5 (10.4)	18 (47.9)	10 (20.8)	13 (27.1)
Only abnormal sensory conduction studies *N* = 7	0	1 (14.3)	1 (14.3)	1 (14.3)
Abnormal motor / sensory conduction studies *N* = 116	27 (23.3)	33 (28.4)	20 (17.2)	30 (25.9)

*FP, fibrillation potentials; PSW, positive sharp waves; MUAPs, motor unit action potentials*.

Nine arms (11.8%) with normal NCS showed abnormal MUAP configuration. Isolated increased polyphasic MUAPs were found in four arms, isolated long duration MUAPS in two arms, isolated high amplitude MUAPS in two arms, and abnormal amplitude and duration in one arm (Table 2). No abnormal spontaneous muscle fiber activity was found in any of these cases. All of these patients had further examinations, which did not reveal any electrophysiological or ultrasound-supported diagnosis UNE. Four patients underwent ultrasounds of the ulnar nerve, in four patients (four arms) the neurologist asked for an additional NCS/EMG examination for differential diagnostics, and three patients underwent an MRI of the cervical spine. In spite of negative repeated neurophysiological examinations (both NCS/EMG and ultrasound), one patient underwent surgical treatment for UNE. Four patients were not diagnosed with a neurological disorder and got a “wait-and-see” advice. Two patients were diagnosed with CTS, one patient with C8 radiculopathy, and one patient with Neuralgic Amyotrophy.

In four patients all SNAP and CMAP responses were absent. Therefore, localization of the ulnar nerve lesion was not possible. Three of these patients had abnormal spontaneous muscle fiber activity. All of these patients underwent an ultrasound examination of the ulnar nerve to localize the lesion, three of them had UNE. One patient had an ulnar nerve entrapment at the wrist and forearm.

## Discussion

This study shows that in our patients, if NCS were normal, no abnormal spontaneous muscle fiber activity was found with EMG. However, in nine patients (11.8%) abnormal configuration MUAPs were found even though NCS were normal. These findings should be interpreted carefully. Abnormal MUAP configuration MUAPs may be indicative of UNE without nerve conduction slowing, UNE in the past or a lesion at another level/site, such as C8 radiculopathy. The interpretation of abnormal MUAP configuration is more at risk for interobserver differences than the interpretation of abnormal spontaneous muscle fiber activity. Operation for UNE will not likely be considered based only on reinnervation characteristics without denervation characteristics. Regardless of its lack of specificity for the diagnosis UNE, abnormal MUAP configurations are important to further investigate differential diagnoses.

In the diagnostic setting with a high prior-odds of UNE (12), the investigator could consider to refrain from EMG for exclusive confirmation of the clinical diagnosis UNE, however in order to evaluate the differential diagnosis of radiculopathy, plexopathy or other neuropathies, needle EMG has added value. Since EMG is often the most painful part of the examination, patients benefit from this proposed change in protocol. Furthermore, its cost-effectiveness is also important, as disposable EMG needle are expensive. Previous studies which investigated the neurophysiological protocol for the confirmation of UNE also suggested no contribution of EMG to the diagnosis (13–15). However, this was not the focus of these studies. Besides, some of them had only a small sample size. In our study, though, we focused on this subject in a relatively large prospective study group. Electromyography might be relevant to give an indication about the prognosis, but data on this subject in patients with normal NCS in clinically suspected UNE are lacking and should be further investigated. This study was performed to evaluate the protocol exclusively for confirmation of the clinical diagnosis of UNE. When the clinician suspects another etiology in its differential diagnosis an extensive EMG protocol sampling a sufficient amount of muscles is mandatory.

When NCS are abnormal, it could be valuable to perform EMG, as the presence of abnormal spontaneous muscle fiber activity could be associated with a worse outcome or the need to plan treatment rapidly. We did not, however, follow up on our patients to address this point and therefore more research is necessary. If one chooses to perform EMG, it is recommended to examine the FDI as well as the ADM muscle, since in 51.9% (abnormal spontaneous muscle fiber activities) to 66.7% (increased polyphasic MUAPs) of the cases abnormalities are found in only one of two muscles.

We suggest a neurophysiological protocol exclusively for the confirmation of clinical UNE in a high prior-odds diagnostic setting, in which EMG is not standard. EMG has limited value for diagnosis UNE if NCS are normal, but is necessary for evaluating the differential diagnosis.

## Data Availability

The datasets generated for this study are available on request to the corresponding author.

## Author Contributions

JS, JM, and FN conceived the study. AK and JS collected the data. AK and FN performed the statistical analyses. AK, JS, WV, JM, and FN reviewed and revised the manuscript for intellectual content.

### Conflict of Interest Statement

The authors declare that the research was conducted in the absence of any commercial or financial relationships that could be construed as a potential conflict of interest.

## References

[1] Bartels RH Menovsky T Van Overbeeke JJ Verhagen WI. Surgical management of ulnar nerve compression at the elbow: an analysis of the literature. J Neurosurg. (1998) 89:722–7. 10.3171/jns.1998.89.5.0722 9817408

[2] Latinovic R. Incidence of common compressive neuropathies in primary care. J Neurol Neurosurg Psychiatry. (2006) 77:263–5. 10.1136/jnnp.2005.066696 16421136 PMC2077603

[3] Mondelli M Giannini F Ballerini M Ginanneschi F Martorelli E. Incidence of ulnar neuropathy at the elbow in the province of Siena (Italy). J Neurol Sci. (2005) 234:5–10. 10.1016/j.jns.2005.02.010 15993135

[4] Bartels RHMA Verhagen WIM van der Wilt GJ Meulstee J van Rossum LGM Grotenhuis JA. Prospective randomized controlled study comparing simple decompression versus anterior subcutaneous transposition for idiopathic neuropathy of the ulnar nerve at the elbow: part 1. Neurosurgery. (2005) 56:522–30. 10.1227/01.NEU.0000154131.01167.03 15730578

[5] Claes F Verhagen WIM Meulstee J. Current practice in the use of nerve conduction studies in carpal tunnel syndrome by surgeons in the Netherlands. J Hand Surg Eur Vol. (2007) 32:663–7. 10.1016/J.JHSE.2007.09.007 17993428

[6] Tamura A Sonoo M Hoshino S Iwanami T Shimada H Miki T. Stimulus duration and pain in nerve conduction studies: stimulus duration and pain. Muscle Nerve. (2013) 47:12–6. 10.1002/mus.23446 23042136

[7] Alshaikh NM Martinez JP Pitt MC. Perception of pain during electromyography in children: a prospective study: pain during pediatric EMG. Muscle Nerve. (2016) 54:422–6. 10.1002/mus.25069 26852012

[8] Strommen JA Daube JR. Determinants of pain in needle electromyography. Clin Neurophysiol. (2001) 112:1414–8. 10.1016/S1388-2457(01)00552-1 11459681

[9] AAEM Quality Assurance Committee Practice Parameter for electrodiagnostic studies in ulnar neuropathy at the elbow. guidelines in electrodiagnostic medicine (2015). update from: AAEM, practice parameter for electrodiagnostic studies in ulnarneuropathy at the elbow, summary statement. Muscle Nerve. (1999) 22:408–11. 10086904 10.1002/(sici)1097-4598(199903)22:3<408::aid-mus16>3.0.co;2-7

[10] van Dijk JG Meulstee J Zwarts M Spaans F. What is the best way to assess focal slowing of the ulnar nerve? Clin Neurophysiol. (2001) 112:286–93. 10.1016/S1388-2457(00)00549-6 11165531

[11] Kasius KM Riphagen JH Verhagen WIM Meulstee J. An easily applicable alternative method for warming cold limbs in nerve conduction studies. Neurophysiol Clin. (2014) 44:219–26. 10.1016/j.neucli.2014.03.001 24930944

[12] Fuller G. How to get the most out of nerve conduction studies and electromyography. J Neurol Neurosurg Psychiatry. (2005) 76(Suppl. 2):ii41–6. 10.1136/jnnp.2005.067355 15961868 PMC1765696

[13] Logigian EL Villanueva R Twydell PT Myers B Downs M Preston DC. Electrodiagnosis of ulnar neuropathy at the elbow (UNE): a Bayesian approach. Muscle Nerve. (2014) 49:337–44. 10.1002/mus.23913 23716377

[14] Omejec G Podnar S. Proposal for electrodiagnostic evaluation of patients with suspected ulnar neuropathy at the elbow. Clin Neurophysiol. (2016) 127:1961–7. 10.1016/j.clinph.2016.01.011 26971477

[15] Todnem K Michler RP Wader TE Engstrøm M Sand T. The impact of extended electrodiagnostic studies in ulnar neuropathy at the elbow. BMC Neurol. (2009) 9:52. 10.1186/1471-2377-9-52 19814833 PMC2767342

